# Fracture Mechanical Properties of Double-Edge Cracked Flattened Brazilian Disc Samples Under Compressive Loads

**DOI:** 10.3390/ma18040850

**Published:** 2025-02-15

**Authors:** Wen Hua, Wenyu Zhang, Shiming Dong, Jianxiong Li, Jiuzhou Huang, Ping Luo, Zhanyuan Zhu

**Affiliations:** 1 College of Mechanical and Electrical Engineering, Xichang University, Xichang 615000, China; huawen_hw@126.com (W.H.);; 2 Failure Mechanics and Engineering Disaster Prevention, Key Laboratory of Sichuan Province, Sichuan University, Chengdu 610065, China; 3 College of Resources and Environment, Xichang University, Xichang 615000, China; 4 School of Automation, Chengdu University of Information Technology, Chengdu 610225, China

**Keywords:** compression-shear loading, true mode II fracture, double-edge cracked flattened Brazilian disc, stress intensity factors, T-stress

## Abstract

The shear-based fracturing of deep fractured rocks under compression-shear loading is one of the most prevalent failure modes due to the existence of in situ stress. In order to study the shear fracture mechanical properties of fractured rocks, a double-edge cracked flattened Brazilian disc (DCFBD) sample was developed by introducing two platforms into a double-edge cracked Brazilian disc (DCBD). Extensive finite element analyses were conducted on DCFBD samples to determine the stress intensity factors (SIFs) and T-stress. A comprehensive dataset of SIFs and T-stress was obtained, which provided accurate descriptions of the compression-shear fracture tests performed on this specimen. Furthermore, the effects of the load distribution angle *γ*, dimensionless crack length *α*, and crack inclination angle *θ* on dimensionless SIFs *Y*_I_, *Y*_II_, and T-stress T* were discussed. It showed that the effect of load distribution angle *γ* on the dimensionless SIFs *Y*_I_ and *Y*_II_ can be disregarded when the dimensionless crack length *α* ≥ 0.60 and load distribution angle *γ* ≤ 20°. However, it should be considered for the T-stress for larger crack inclination angles. Moreover, it was experimentally validated that the DCFBD samples with appropriate crack lengths and load distribution angles can achieve shear (true mode II) fracture, as demonstrated through a series of fracture tests conducted on these specimens. The results will advance the development of rock shear fracture testing technology.

## 1. Introduction

Fractured rocks in various rock engineering practices, such as underground mining, oil and gas exploration, nuclear waste disposal, etc., are usually subjected to compression-shear stress states [1,2,3,4,5]. Among various failure modes in rock engineering, the most prevalent is the shear sliding of deep fractured rocks subjected to compression-shear loading [6,7,8,9]. Furthermore, the occurrence of many geological disasters, such as landslides and collapses, is primarily attributed to the shear sliding of fractured rock. Hence, it is crucial to study the fracture characteristics of rocks under compression-shear loading for the assessment of rock structural stability and rock engineering safety [9,10,11,12].

**Figure 1:**
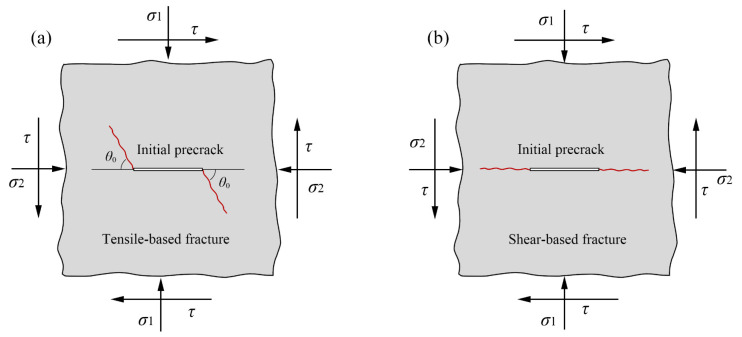
Schematic diagram of fracture mode of fractured rocks under compression-shear loading [24]. (**a**) Tensile-based fracture; (**b**) Shear-based fracture.

**Figure 2:**
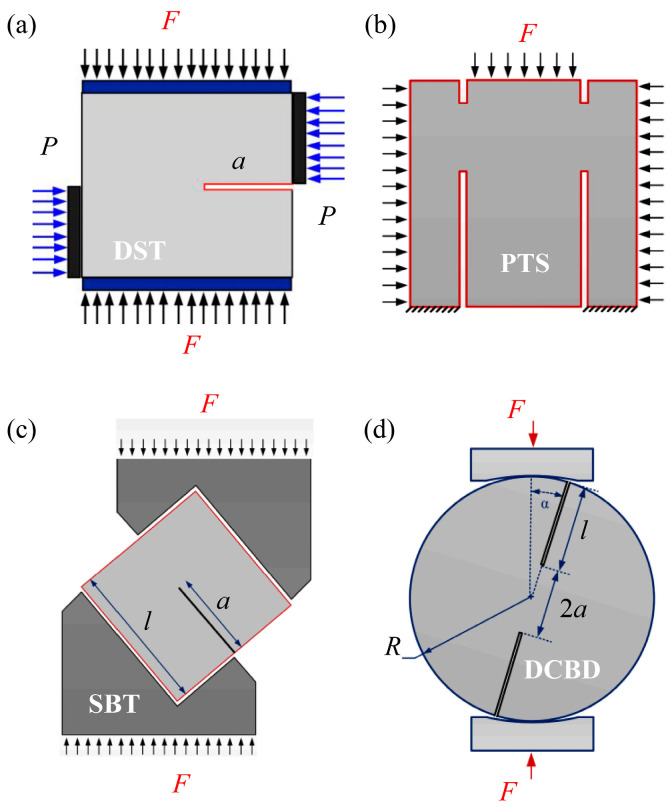
Shear-based (true mode II) fracture samples under compression-shear loading. (**a**) DST sample, (**b**) PTS sample, (**c**) SBT sample, (**d**) DCBD sample.

For the purpose of investigating the fracture mechanical characteristics of fractured rocks under compression-shear loading, scholars have proposed many cracked sample configurations such as the centrally cracked Brazilian disc sample [13,14,15,16], inclined cracked rectangular plate sample [5,17], internally cracked plate sample [6,18,19], antisymmetric four-point bending sample [20], etc. Although these aforementioned cracked configurations can achieve compression-shear loading, they still exhibit a dominant fracture mode characterized by tensile failure. The reason for this is that the tensile strength of rocks is considerably inferior to their shear strength, resulting in tensile failure occurring prior to shear failure [21]. The failure modes of fractured rocks under compression-shear loading exhibit two typical fracture modes: tensile-based (opening crack, mode I) fracture and shear-based (shear sliding, self-similar plane expansion, true mode II) fracture. These two fracture modes are fundamentally distinct from each other, as illustrated in Figure 1. The effective inhibition of crack tip tensile stress is crucial for achieving shear-based fracture in fractured rocks [22,23,24]. In recent years, scholars have proposed several various cracked configurations (see Figure 2) that can facilitate rock shear-based fracture, providing an effective approach for measuring rock shear (true mode II) fracture toughness.

The direct shear test (DST, Figure 2a) was originally mainly used to measure the shear strength of rocks, and then gradually developed into a testing method to study the shear (true mode II) fracture mechanical properties of fractured rocks [25]. However, this method requires two sets of independent loading systems, which requires a high flatness of the samples. Moreover, when the vertical pressure is small, wing cracks caused by tensile stress will be generated, and self-similar plane expansion (shear-based fracture) cannot be realized. Backers et al. [26] proposed a punch-through shear (PTS) sample (Figure 2b) for conducting rock shear fracture tests, which was subsequently suggested by the International Society for Rock Mechanics (ISRM) as the standardized approach for measuring rock shear (true mode II) fracture toughness [27]. However, this method requires an additional confining pressure loading device, and the confining pressure size is also required. Low confining pressure can produce wing cracks caused by tensile stress, while high confining pressure can effectively inhibit crack tip tensile stress to achieve shear-based fracture, but it will further increase the friction of the crack surface, thus increasing the tested shear (true mode II) fracture toughness value. In addition, due to the small crack tip ligaments in this sample, the measured shear fracture toughness may be underestimated for rock materials with a large fracture process zone [22]. Yao et al. [28] further extended this PTS sample to rock dynamic fracture tests. It was found that similar to under static loading, wing cracks caused by tensile stress were also generated under dynamic loading with low confining pressure. Additionally, Rao et al. [29] employed a shear-box testing method (SBT, as depicted in Figure 2c) to conduct shear fracture tests of rocks. It requires only one set of loading device; however, the shear box must be customized with different angles according to the actual dimensions of the cracked samples. Moreover, high precision is necessary for sample processing, rendering it impractical for routine use.

In addition, Chen et al. [30,31,32] firstly proposed a DCBD sample, as shown in Figure 2d, to study the compression-shear fracture mechanical properties of rocks. The SIFs around the crack tip in DCBD samples under compression have been calculated using various methods [22,33,34,35,36]. Furthermore, recently, our team derived the computational formulae for SIFs of DCBD samples under different distribution loads, highlighting that the effect of load distribution angle on SIFs is negligible for DCBD samples with larger, dimensionless crack lengths [23,24,35]. Moreover, a series of experimental investigations have been conducted using DCBD samples made from various kinds of rocks, showing that the shear fracture mode can also be achieved by appropriately selecting the crack length and crack inclination angle in DCBD samples [23,24,37,38]. In comparison to the aforementioned cracked configurations, the DCBD sample offers several advantages such as closed-form solutions for fracture parameters at the crack tip, ease of processing, and no need for additional confining pressure loading devices or special shear boxes. Furthermore, conventional presses can be used for loading. Moreover, Bahrami et al. [22] demonstrated that the DCBD sample has the least inhibition impact on crack tip tensile stress compared to the PTS and DST samples, making it more likely to obtain the shear fracture toughness. However, Tang et al. [23] indicated that achieving shear-based fracture in DCBD samples loaded with flat platens or curved jaws is not feasible. In order to achieve shear-based fracture of rocks, it is necessary to utilize two plastic cushions or wood blocks to mitigate intensive stress concentration at the contact area between the DCBD sample and loading devices [4,22,23,39]. It is worth noting that the introduction of two platforms into the Brazilian disc or centrally cracked Brazilian disc samples has been also demonstrated to effectively mitigate stress concentration, as evidenced by Wang et al. [40,41,42]. Moreover, Yan et al. [4] recently also introduced two platforms into the DCBD sample, thereby proposing a DCFBD sample to research the dynamic true mode II fracture characteristics of rocks. However, their study only considered DCFBD samples with 0.65 ≤ *α* ≤ 0.85. In addition, the crack inclination angles in their study were equal to half of the load distribution angle and both fell within the range of 10° ≤ *θ* ≤ 15°. Hence, the available fracture parameters such as SIFs and T-stress in DCFBD samples are very limited, and these samples lack systematic research. The effects of load distribution angle, crack length, and crack inclination angle on the SIFs and T-stress of these samplse remain unclear. Therefore, it is imperative to conduct a comprehensive investigation on SIFs and T-stress of these samples. Meanwhile, it is also very important to conduct experimental studies to verify the shear (true mode II) fracture capability of DCFBD samples.

In this work, a systematic analysis of DCFBD samples was carried out to investigate the shear fracture properties. Firstly, extensive finite element analyses were conducted on DCFBD samples subjected to uniform pressure to determine the SIFs and T-stress around the crack tip using the finite element method (FEM). A comprehensive dataset of SIFs and T-stress was obtained, which provided accurate descriptions of the compression-shear fracture tests performed on the samples. Subsequently, the impacts of load distribution angle, dimensionless crack length, and crack inclination angle on dimensionless SIFs and T-stress were systematically discussed. Moreover, a series of fracture experiments were conducted on DCFBD samples to verify their shear (true mode II) fracture capability. The results will advance the development of rock shear fracture testing technology.

## 2. SIFs and T-Stress for DCFBD Samples with Finite Element Method

**Figure 3:**
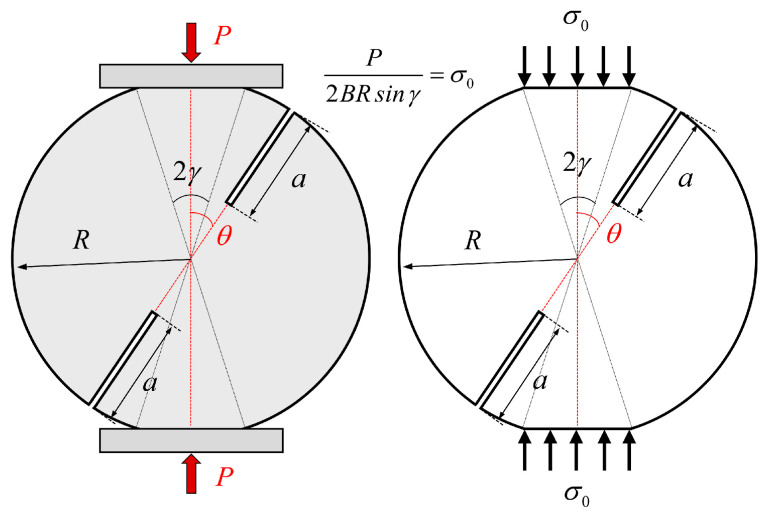
Schematic diagram of DCFBD sample under uniform distribution pressure.

In order to establish a fracture test method based on DCFBD samples for investigating the fracture characteristics of rock masses under compression-shear loading, it is crucial to accurately compute the SIFs and T-stress around the crack tip [34,43,44,45,46]. Several different methods have been extensively utilized to obtain the SIFs and T-stress, including the weight function method [34,47], boundary collocation method [33], complex function method [48], phase field method [49], FEM [22,36], etc. In this work, the finite element method was employed for calculating SIFs and T-stress in DCFBD samples. Figure 3 shows a DCFBD sample with a radius of *R*, a thickness of *B*, and an edged-crack length of *a*, which was subjected to the uniform pressure *σ*_0_ along the contact zone with a load distribution angle of 2*γ*. The angle *θ* between the prefabricated crack and the loading direction is called the crack inclination angle. By considering the equilibrium relationship, one can readily establish the correlation between the resultant force *P* and the uniform pressure *σ*_0_ as follows:

(1) $$\sigma_{0}=\frac{P}{2BR\sin\gamma }$$

Similar to that of the DCBD samples [22,23,35], the mode I and mode II SIFs *K*_I_, *K*_II_, and T-stress *T* for the DCFBD samples are expressed as:

(2) $$\left\{\begin{array}{l} K_{I}=\frac{P}{\pi BR}\sqrt{\pi a}\cdot Y_{I}\left(\alpha ,\theta ,\gamma \right)\\ K_{\mathrm{II}}=\frac{P}{\pi BR}\sqrt{\pi a}\cdot Y_{\mathrm{II}}\left(\alpha ,\theta ,\gamma \right)\\ T=\frac{P}{\pi BR}\cdot T*\left(\alpha ,\theta ,\gamma \right) \end{array}\right.$$

where *α* = *a*/*R* is the dimensionless crack length. *Y*_I_ and *Y*_II_ are the dimensionless mode I and mode II SIFs, respectively, and T* is the dimensionless T-stress. These dimensionless values are only dependent on sample geometry and crack inclination angle (i.e., *α*, *θ*, *γ*) and independent of external load *P* [50]. The SIFs *K*_I_, *K*_II_, and T-stress *T* around the crack tip of the DCFBD samples were computed using the FEM with ABAQUS2022. A DCFBD finite element model was established by considering a linear elastic and isotropic material with an elastic modulus of 6.70 GPa and Poisson’s ratio of 0.23 [4]. The DCFBD sample was characterized by the following geometrical dimensions: diameter *D* = 100 mm, thickness *B* = 30 mm, load distribution angle *γ* = 5°~20°, dimensionless crack length *α* = 0.40~0.90, and crack inclination angle *θ* = 10°~45°, as listed in Table 1. It should be mentioned that the analyses conducted in our study only considered the case of *γ* ≤ *θ* because of the characteristics of the crack location [23,30].

**Figure 4:**
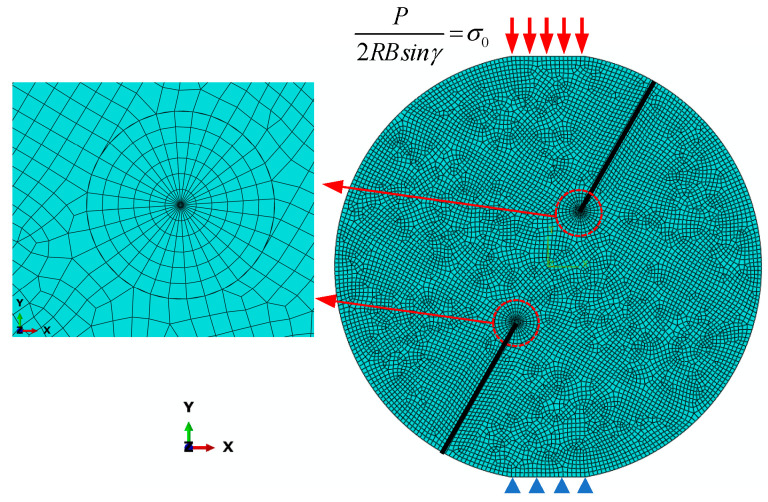
A representative finite element model of the DCFBD sample.

Figure 4 illustrates a representative DCFBD finite element model under uniform pressure. The crack is usually regarded as an ideal Griffith crack, which is assumed to have zero width. In the vicinity of crack tips, concentric circles were utilized for meticulous mesh division, with a total of six contours. The interior of the contours was divided into grids using a sweep technique, while the exterior of the contours was meshed using free grid meshing technology. The CPS6M element was used in the inner rings surrounding the crack tip, while other parts were considered as CPS8R elements. Furthermore, the 1/4 nodal singular elements were employed to accurately describe the stress singularity around the crack tip [51]. The displacement boundary of the bottom platform of the DCFBD finite element model was fixed. A uniform pressure *σ*_0_ = 1.00 MPa was applied to the upper platform of the DCFBD model. It should be noted that the finite element model does not account for contact interactions and frictional effects between crack surfaces. The SIFs and T-stress were directly computed with the integration method in ABAQUS software.

In order to validate our finite element model, we performed a comparison of the values reported by Yan et al. [4]. Yan et al. [4] also computed the SIFs and T-stress in DCFBD samples with 0.65 ≤ *α* ≤ 0.85. In addition, the crack inclination angles in their study were equal to half of the load distribution angle and both fell within the range of 10° ≤ *θ* ≤ 15°. The values of *Y*_I_, *Y*_II_, and T* were recalculated using Equation (2) with the values of *K*_I_, *K*_II_, and *T* reported by Yan et al. [4]. The recalculated dimensionless SIFs *Y*_I_ and *Y*_II_ as well as the dimensionless T-stress T* are presented in Table 2. In addition, the values obtained from the finite element model developed in this work are also presented in Table 2. As shown in Table 2, the comparison reveals a high level of agreement between our calculations and theirs, indicating the excellent accuracy of our finite element models.

## 3. Finite Element Results for DCFBD Samples

Extensive finite element analyses were carried out on the DCFBD samples, and the dimensionless values of SIFs and T-stress in the DCFBD samples with varying dimensionless crack lengths, crack inclination angles, and load distribution angles were calculated using Equation (2) based on the finite element results. In this section, the impacts of load distribution angle, dimensionless crack length, and crack inclination angle on dimensionless SIFs and T-stress are systematically discussed.

### 3.1. Dimensionless Mode I SIF

**Figure 5:**
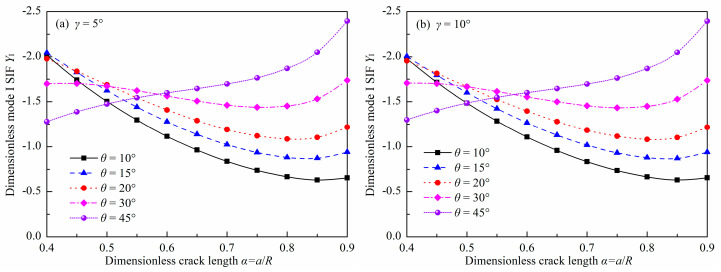

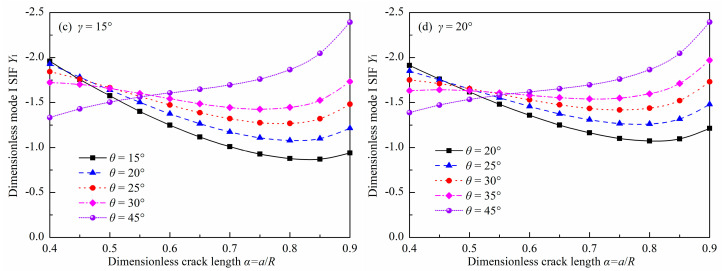
Values of *Y*_I_ in the DCFBD samples: (**a**) *γ* = 5°, (**b**) *γ* = 10°, (**c**) *γ* = 15°, (**d**) *γ* = 20°.

**Figure 6:**
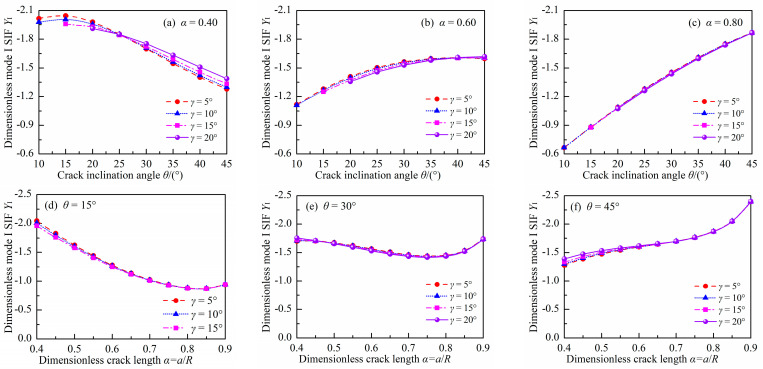
Values of *Y*_I_ in the DCFBD samples: (**a**) *α* = 0.40, (**b**) *α* = 0.6, (**c**) *α* = 0.80, (**d**) *θ* = 15°, (**e**) *θ* = 30°, (**f**) *θ* = 45°.

Figure 5 and Figure 6 show the values of *Y*_I_ for the DCFBD samples with varying load distribution angles, dimensionless crack lengths, and crack inclination angles under uniform pressure. As shown in Figure 5 and Figure 6, the values of *Y*_I_ are consistently negative. In fact, the issue of contact between crack surfaces has not been taken into account in the finite element models. Consequently, the values of *Y*_I_ can be considered to be negative. The negative values indicate that the crack is subjected to compressive stress, leading to a tendency for the crack surfaces to close. This phenomenon is contrary to that of an opening crack. Moreover, the absolute values can indirectly represent the magnitude of the compressive stress. It indicates that the DCFBD sample cannot realize tensile-shear loading [23,52]. On the contrary, this kind of sample is very appropriate for studying the fracture behaviors of rocks subjected to compression-shear loading [22,23,32,39].

It can be also found from Figure 5 and Figure 6 that the effect of *α* on *Y*_I_ is intricate. As *α* increases, the absolute value of *Y*_I_ decreases and then increases when *θ* ≤ 30°. Nevertheless, its absolute value for *θ* = 45° always increases with increasing *α*, as presented in Figure 6f. In addition, Figure 5 also shows a decrease in the absolute value of *Y*_I_ as *θ* increases for a small *α* (i.e., *α* = 0.40). Nevertheless, when *α* ≥ 0.60, the absolute value of *Y*_I_ increases as *θ* increases. For example, the absolute values of *Y*_I_ for the DCFBD sample with *α* = 0.40 and *γ* = 15°, at crack inclination angles of 15°, 30°, and 45°, are 1.959, 1.724, and 1.333, respectively. These values are decreased by 12% and 32% when compared to the crack inclination angle of 15°. However, for *α* = 0.70 and *γ* = 15°, when the crack inclination angles are 15°, 30°, and 45°, the absolute values of *Y*_I_ are 1.010, 1.444, and 1.696, respectively. These values exhibit an increase of 43% and 68% compared to that of a crack inclination angle at 15°. This phenomenon is completely consistent with the DCBD samples [23,35]. Bahrami et al. [22] indicated that a smaller crack inclination angle may be more appropriate for carrying out shear-based fracture experiments with DCBD samples. To minimize the impact of negative *Y*_I_ on the shear fracture toughness of DCFBD samples, it is necessary to employ a greater value of *α* and a smaller value of *θ*.

Figure 6 also shows the impact of *γ* on *Y*_I_ for several typical cases. It indicates that the impact of *γ* on *Y*_I_ is intensively associated with the values of *α* and *θ*. Notably, a significant influence of *γ* on *Y*_I_ is observed when *α* has a smaller value (i.e., *α* = 0.40), as depicted in Figure 6a. However, once *α* exceeds 0.60, the impact of *γ* on *Y*_I_ becomes small, with its degree decreasing as *α* increases. For instance, the *Y*_I_ value of the DCFBD sample with *α* = 0.40 changes from −2.045 to −1.959 for *θ* = 15°, and from −1.279 to −1.333 for *θ* = 45° as the *γ* changes from 5° to 15°. For the DCFBD sample with *α* = 0.60, *Y*_I_ varies from −1.278 to −1.249 for *θ* = 15°, and from −1.598 to −1.607 for *θ* = 45° when *γ* changes from 5° to 15°. The further analysis reveals that the *Y*_I_ value in DCFBD samples with *α* ranging from 0.40 to 0.90 exhibits a maximum relative change of approximately 4.2% when there is a variation in *γ* from 5° to 15°. Hence, the effect of load distribution angle *γ* on *Y*_I_ within this range is negligible. However, when *γ* varies from 5° to 20°, there can be a relative change in *Y*_I_ of up to 8.5% for the DCFBD sample with *α* = 0.40. Therefore, it becomes imperative to consider the impact of the load distribution angle at this point.

### 3.2. Dimensionless Mode II SIF

**Figure 7:**
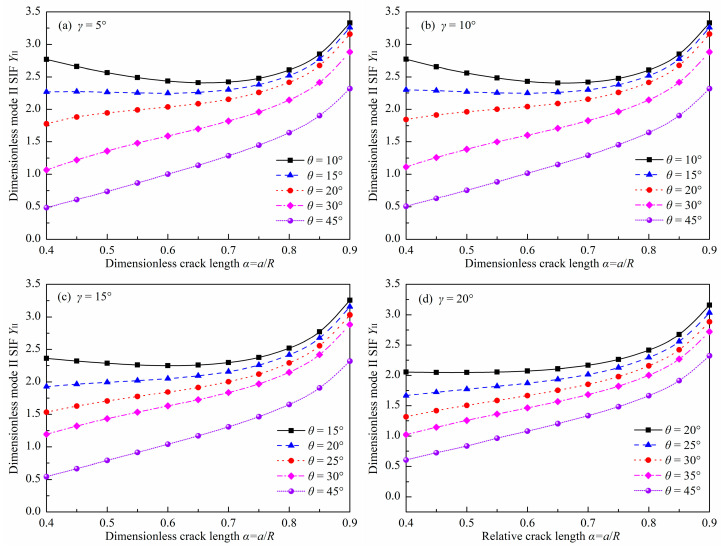
Absolute values of *Y*_II_ in the DCFBD samples: (**a**) *γ =* 5°, (**b**) *γ =* 10°, (**c**) *γ =* 15°, (**d**) *γ =* 20°.

**Figure 8:**
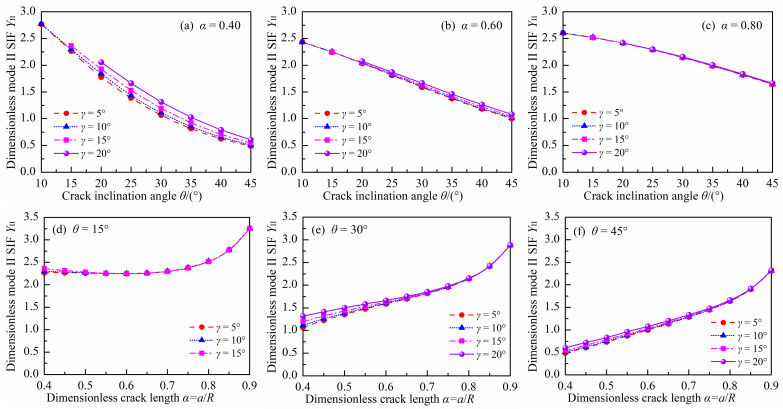
Absolute values of *Y*_II_ in the DCFBD samples: (**a**) *α* = 0.40, (**b**) *α* = 0.6, (**c**) *α* = 0.80, (**d**) *θ* = 15°, (**e**) *θ* = 30°, (**f**) *θ* = 45°.

Figure 7 and Figure 8 show the absolute values of *Y*_II_ for the DCFBD samples with different values of *γ*, *α,* and *θ* under uniform pressure. It is noteworthy that the *Y*_II_ of the DCFBD samples, as depicted in Figure 3 and Figure 4, also exhibits a negative sign. Tang et al. [23] have emphasized that the negative/positive *Y*_I_ signifies crack surface closing/opening, while the negative/positive *Y*_II_ solely expresses the direction of shear fracture. Hence, the magnitude (absolute value) of *Y*_II_ is plotted in Figure 7 and Figure 8, and subsequently, our discussion focuses on its magnitude.

Figure 7 also indicates that the impact of *α* on *Y*_II_ is found to be dependent on the value of *θ*. When *θ* = 10°, *Y*_II_ initially decreases and then increases with increasing *α*, as presented in Figure 7a,b. However, when *θ* exceeds 15°, its value consistently increases with increasing α. Furthermore, it is observed that *Y*_II_ always decreases with increasing *θ* for given values of *α* and *γ*, as depicted in Figure 8a–c. This behavior differs from that of *Y*_I_, as presented in Figure 5 and Figure 6. For instance, the values of *Y*_II_ for the DCFBD sample with *α* = 0.40 and *γ* = 15°, at *θ* = 15°, 30°, and 45°, are respectively 2.361, 1.194, and 0.543. These values are decreased by 49% and 77% when compared to the case of θ = 15°. However, when *γ* = 15° and *α* = 0.70, the value of *Y*_II_ for the DCFBD sample with *θ* = 15°, 30°, and 45°, are respectively calculated as 2.296, 1.835, and 1.308. These values show a reduction of 20% and 43% in comparison to that obtained for *θ* = 15°. Tang et al. [23] and Bahrami et al. [22] reported that achieving shear-based fracture of the DCBD samples requires a relatively large mode II SIF *Y*_II_. Therefore, it is also crucial to have a relatively long crack length and a small crack inclination angle to achieve the shear-based fracture of the DCFBD samples under compression-shear loading.

In addition, the impact of *γ* on *Y*_II_ is illustrated in Figure 8. As shown in Figure 8, the impact of *γ* on *Y*_II_ is found to be dependent on the values of *α* and *θ*. For a smaller α value (i.e., *α* = 0.40), there is a significant impact of *γ* on *Y*_II_. However, when α exceeds 0.60, the impact of *γ* on *Y*_II_ can be neglected. For example, in a DCFBD sample with *α* = 0.40, the value of *Y*_II_ exhibits an increase from 2.266 to 2.361 for *θ* = 15°, and from 0.486 to 0.543 for *θ* = 45° when *γ* changes from 5° to 15°, respectively. Furthermore, when *γ* = 20°, there can be a relative increase in *Y*_II_ of up to 24% for the DCFBD sample with *α* = 0.40. Therefore, the impact of *γ* on *Y*_II_ must be considered for the DCFBD sample with small crack lengths. However, in the case of the DCFBD with *α* = 0.60, *Y*_II_ varies from 2.246 to 2.249 for *θ* = 15°, and fluctuates between 1.001 and 1.040 for *θ* = 45° when altering *γ* from 5° to 15°. These results demonstrate that the value of *Y*_II_ for the DCFBD sample with *α* = 0.60 exhibits a maximum relative change of only approximately 4.0% when varying *γ* from 5° to 15°. Further investigation reveals that the maximum disparity in *Y*_II_ of the DCFBD samples with *α* ≥ 0.70 between *γ* = 5° and *γ* = 20° is approximately 3.8%, indicating that the impact of *γ* on *Y*_II_ can be disregarded. Hua and his co-works [23,35] demonstrated that the impact of *γ* on *Y*_II_ for the DCBD samples is negligible when *α* exceeds 0.60. It is evident that the conclusion obtained in this study for the DCFBD samples is in perfect agreement with that reported by Hua et al. [23,35] for the DCBD samples.

### 3.3. Dimensionless T-Stress

**Figure 9:**
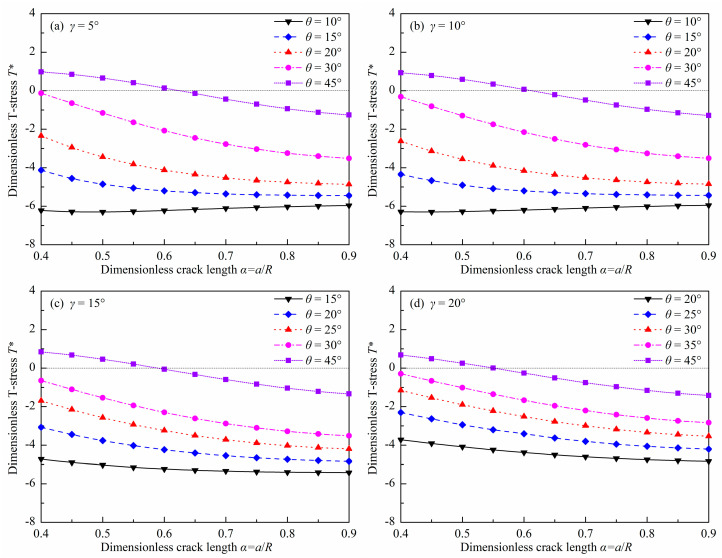
Dimensionless T-stress T* of the DCFBD samples: (**a**) *γ* = 5°, (**b**) *γ* = 10°, (**c**) *γ* = 15°, (**d**) *γ* = 20°.

**Figure 10:**
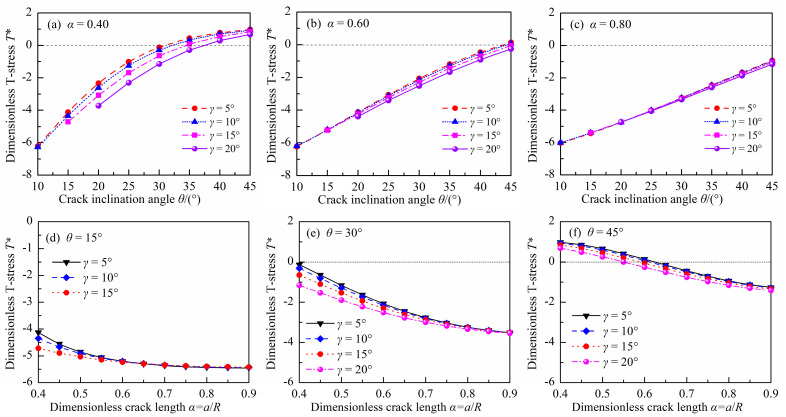
Dimensionless T-stress T* of the DCFBD samples: (**a**) *α* = 0.40, (**b**) *α* = 0.6, (**c**) *α* = 0.80, (**d**) *θ* = 15°, (**e**) *θ* = 30°, (**f**) *θ* = 45°.

Figure 9 and Figure 10 show the dimensionless T-stress T* in DCFBD samples with different load distribution angles *γ*, dimensionless crack lengths *α*, and crack inclination angles θ under uniform pressure. As illustrated in Figure 9 and Figure 10, when *θ* ≤ 30° and *α* ≥ 0.4, the T-stress of the DCFBD sample is consistently negative. However, for *θ* = 45°, the T-stress transitions from positive to negative with an increase in the dimensionless crack length *α*, as evident from Figure 10f. Previous studies have shown that both the sign and magnitude of T-stress exert an influence on the crack propagation path and fracture load [53,54,55,56,57,58]. The presence of a negative T-stress consistently leads to a reduction in the initiation angle and an enhancement in fracture toughness. Conversely, positive T-stresses yield opposite effects [43,56,59,60]. Additionally, it is also observed that the dimensionless T-stress T* generally is increased with an increasing *θ*, and is reduced with an increasing α, as presented in Figure 10. The presence of a larger crack length in DCFBD samples always corresponds to a significantly negative T-stress, indicating that as *α* increases, the compressive T-stress becomes more pronounced. Bahrami et al. [22] and Tang et al. [24] showed that a substantial magnitude of compressive (negative) T-stress can effectively inhibit crack kinking, thereby promoting the occurrence of shear-based fracture.

Furthermore, Figure 10 also show the impact of *γ* on T*. It can be observed that the impact of *γ* on T* is also closely related to the values of *α* and *θ*. For a smaller *θ* (i.e., *θ* = 15°), the value of *γ* significantly influences the dimensionless T-stress T* for the DCFBD sample with a small crack length. However, when *α* exceeds 0.60, the effect of *γ* on T* can be disregarded, as depicted in Figure 10d. For example, the value of T* in the DCFBD sample with *α* = 0.40 undergoes a change from −4.125 to −4.718 for *θ* = 15° as *γ* varies from 5° to 15°. However, when considering the DCFBD with *α* = 0.60, T* only varies from −5.200 to −5.232 for *θ* = 15° when altering *γ* from 5° to 15°. Nevertheless, Figure 10f indicates that there is a significant impact of *γ* on T* for the DCFBD sample with a larger *θ* (i.e., *θ =* 45°) even in cases where crack lengths are large. Changing *γ* from 5° to 15°, the value of T* for the DCFBD sample with *α* = 0.40 decreases by 14%, changing from 0.977 to 0.841 for *θ* = 45°. In the case of the DCFBD with *α* = 0.60, altering *γ* from 5° to 15° leads to a reduction of T* by 40% from 0.139 to −0.056 for *θ* = 45°. This indicates that the impact of *γ* on T* for the DCFBD sample cannot be disregarded, which differs from its impact on SIFs. Hence, it can be concluded that T-stress is more sensitive to the load distribution angle than SIFs.

## 4. Fracture Experiments

The compression-shear fracture experiments were conducted on DCFBD samples fabricated from a green sandstone to validate its capability in achieving true mode II fracture. The selected green sandstone, sourced from Ziyang City, Sichuan Province, China, was a representative homogeneous and isotropic material with an approximate density of 2.28 g/cm^3^, tensile strength of 5.39 MPa, and Poisson’s ratio of 0.23. The used DCFBD samples had nominal dimensions of 100 mm in diameter and 30 mm in thickness. According to previous experimental findings conducted on DCBD samples [22,23,32,39], our experiments considered the relative crack lengths *α* of 0.6, 0.70, and 0.80, as well as loading angles *θ* of 10°, 15°, 20°, and 25°. Additionally, this study exclusively focused on cases where the loading angle *θ* was equivalent to the load distribution angle *γ*, as utilized by Yan et al. [4]. The DCFBD samples with the aforementioned dimensions were fabricated from sandstone slabs using a computer-controlled water jet cutting machine. The measured notch width in these DCFBD samples was approximately 1.50 mm. The fracture tests were performed with an electro-hydraulic servo electronic universal material testing machine (DF13.305D). The loading process was carried out under displacement control with a displacement loading rate of 0.10 mm/min to satisfy the requirements of static loading conditions [61,62,63]. During the tests, two polymethyl methacrylate cushions were inserted between the DCFBD sample and loading devices [22,23]. The DCFBD samples underwent loading until final failure, with comprehensive load–displacement data being recorded by the testing system throughout each test.

**Figure 11:**
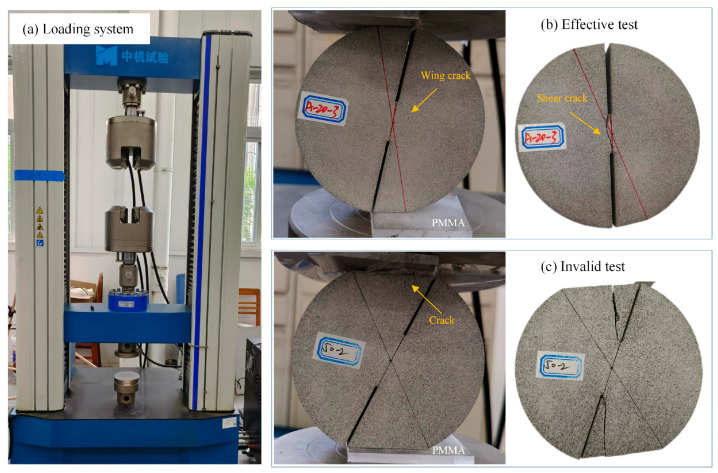
The representative loading diagram of DCFBD samples under compression.

**Figure 12:**
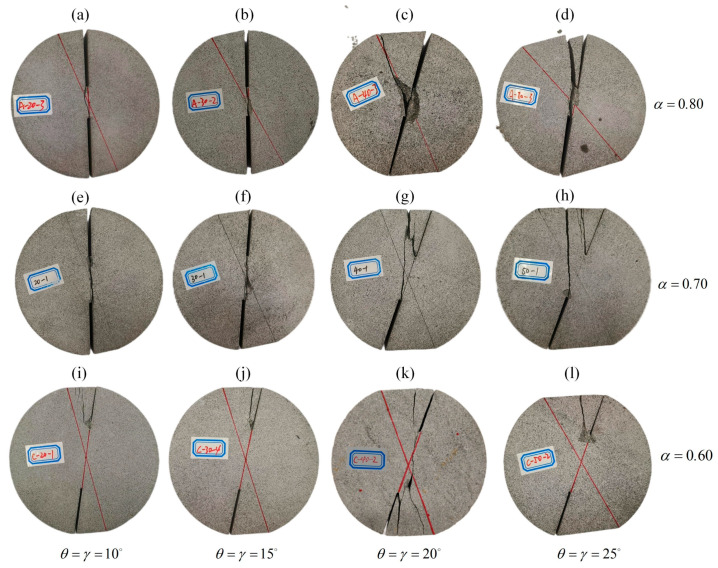
Fracture forms of DCFBD samples with different loading angles and crack lengths. (**a**) *α* = 0.80, *θ* = *γ* = 10°; (**b**) *α* = 0.80, *θ* = *γ* = 15°; (**c**) *α* = 0.80, *θ* = *γ* = 20°; (**d**) *α* = 0.80, *θ* = *γ* = 25°; (**e**) *α* = 0.70, *θ* = *γ* = 10°; (**f**) *α* = 0.70, *θ* = *γ* = 15°; (**g**) *α* = 0.70, *θ* = *γ* = 20°; (**h**) *α* = 0.70, *θ* = *γ* = 25°; (**i**) *α* = 0.60, *θ* = *γ* = 10°; (**j**) *α* = 0.60, *θ* = *γ* = 15°; (**k**) *α* = 0.60, *θ* = *γ* = 20°; (**l**) *α* = 0.60, *θ* = *γ* = 25°.

Figure 11 and Figure 12 illustrate the representative loading diagrams and the fracture forms of the DCFBD samples under compressive loads. For a small loading angle (i.e., *θ* = 10°), as presented in Figure 11b, a tensile wing crack emerges on the specimen surface upon application of the load, followed by subsequent propagation of shear cracks along the direction of the pre-existing crack, which aligns with the definition of true mode II (shear-based) fracturing. This implies that not only do the stresses around the crack tip satisfy compression-shear loading requirements, but also that the mode of crack propagation exhibits self-similar propagation characteristics [22,23]. However, it has been observed that a vertical crack initially emerges on the upper side of the DCFBD sample with a large loading angle (i.e., *θ* = 25°) during the loading process, as illustrated in Figure 11c. As the load increases, the vertical crack gradually extends upwards until it reaches the crack tip, without undergoing any shear failure at this stage, thereby resulting in an invalid test.

It can be observed from Figure 12 that the DCFBD samples with *θ* = *γ* = 10° and *θ* = *γ* = 15° can effectively achieve true mode II fracture for the two cases of relative crack lengths of 0.70 and 0.80. However, when the relative crack length *α* is 0.60, none of the DCFBD samples are able to achieve shear fracture. This finding is consistent with the results documented by Aminzadeh et al. [64], Tang et al. [23], and Bahrami et al. [22,39], using DCBD samples. For the shear-fractured DCFBD samples, the complete load–displacement curves have been meticulously documented to determine the true mode II fracture toughness.

**Figure 13:**
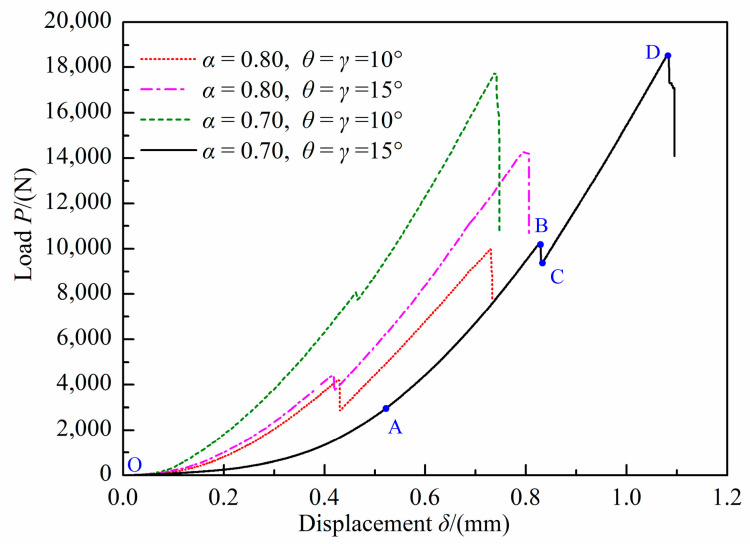
Typical load–displacement curves for the shear fractured DCFBD samples.

Figure 13 presents the representative load–displacement curves of shear fractured DCFBD samples with varying loading angles and crack lengths. As depicted in Figure 13, the load–displacement curve can be categorized into four distinct stages. During the initial loading phase (OA), the concave shape of the load–displacement curve is attributed to the deformations of both polymethyl methacrylate cushions and sandstone specimens. Subsequently, there is a gradual linear increase in load with increasing displacement until reaching point B. Then, there is a sudden slight decrease in load from point B to point C. At this juncture, a tensile wing crack initiates at the crack tip of the sample surface, which can be found in Figure 11b. After that, the load continues to increase linearly with displacement until it reaches point D, where the load reaches its maximum value. During this stage (CD), the tensile wing crack has not yet fully developed, and its propagation ceases. Ultimately, as the shear crack begins to initiate and leads to the abrupt failure of the DCFBD samples, as depicted in Figure 11b and Figure 12a,b,e,f, the load undergoes an almost vertical drop. These findings align perfectly with the load–displacement curves for DCBD samples observed by Hug et al. [65] and Aminzadeh et al. [64]. Moreover, the experimental results obtained by Asadizadeh et al. [66] through the utilization of a digital image correlation technique have indicated that significant shear deformation primarily occurs during the later stages of loading for the DCBD specimens, with a predominant shear failure mode observed in the ligament area. Although the wing crack is observed to initiate from the crack tip prior to the development of the shear crack, it is important to note that the final failure mode of DCFBD samples, as illustrated in Figure 12a,b,e,f, is characterized by shear failure. Additionally, the observed variations in load–displacement curves are in accordance with the predominant fracture characteristics exhibited by brittle or quasi-brittle materials [51,62,67,68]. Therefore, the peak loads of these shear failure samples are considered for determining the true mode II fracture toughness *K*_IIC_ of this sandstone.

**Figure 14:**
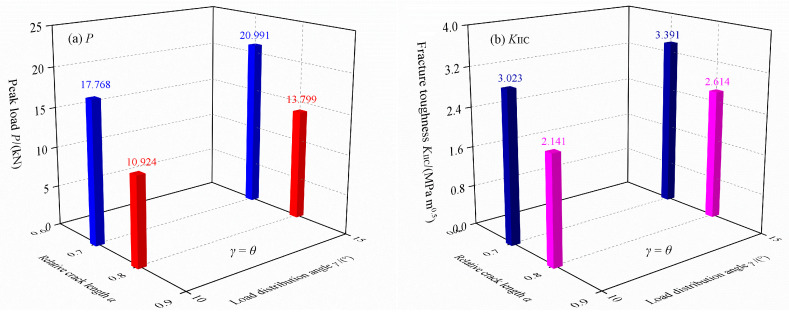
Average tested values of DCFBD specimens: (**a**) peak load *P*, (**b**) true mode II fracture toughness *K*_IIC_.

The fracture tests have been carried out on green sandstone using DCFBD samples, with the experimental data being documented in Table 3. Meanwhile, the average values of peak load and true mode II fracture toughness are also presented in Figure 14. Figure 14a displays that a positive correlation between the load distribution angle of the DCFBD samples and the peak load required for fracture, indicating that an increase in the load distribution angle results in a corresponding increase in peak load for samples with identical crack lengths. Furthermore, for the DCFBD sample with a same loading angle, shorter crack lengths result in greater peak loads. Based on Equation (2), the true mode II fracture toughness *K*_IIC_ has been computed, and the average values are illustrated in Figure 14b. For DCFBD samples with *α* = 0.80, the true mode II fracture toughness *K*_IIC_ for *θ* = *γ* = 10° and 15° are 2.141 and 2.614 MPa m^0.5^, respectively. Nevertheless, the tested values of *K*_IIC_ increase to 3.023 MPa m^0.5^ for *θ* = *γ* = 10° and 3.391 MPa m^0.5^ for *θ* = *γ* = 15°, respectively, when the relative crack length *α* is reduced to 0.70. The results indicate that the tested values of *K*_IIC_ for *α* = 0.70 are approximately 1.35 times higher than those for *α* = 0.80 in both cases of *θ* = *γ* = 10° and 15°. This phenomenon is attributed to the stronger inhibition effect at the crack tip when *α* = 0.70, resulting in relatively larger test values [22].

**Figure 15:**
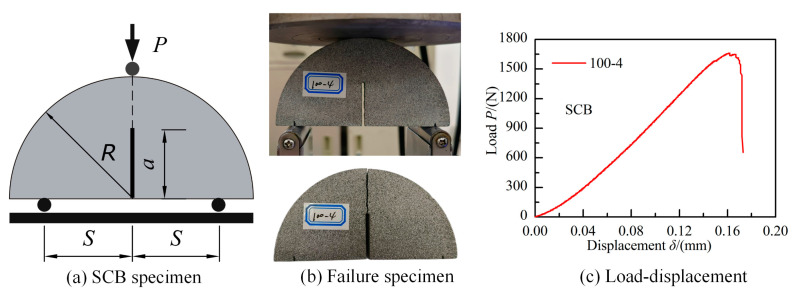
The SCB sample for determining mode I fracture toughness.

In order to compare the values of mode I and mode II fracture toughness (*K*_IC_, *K*_IIC_), the semi-circular bending (SCB) method (Figure 15), recommended by the ISRM [69,70], was employed to determine the mode I fracture toughness of this green sandstone. According to the SCB method [70], the value of *K*_IC_ was computed using the following equation.

(3) $$K_{\mathrm{IC}}=\frac{P_{\max}\sqrt{\pi a}}{2RB}\cdot Y\left(\frac{S}{R},\frac{a}{R}\right)$$

with

(4) $$Y\left(\frac{S}{R},\frac{a}{R}\right)=-1.297+9.516\frac{S}{R}-\left(0.47+16.457\frac{S}{R}\right)\frac{a}{R}+\left(1.071+34.401\frac{S}{R}\right){\left(\frac{a}{R}\right)}^{2}$$

The non-dimensional SIF *Y* was solely determined by the ratio of half span to radius (*S*/*R*) and the relative crack length (*a*/*R*). The SCB specimens made from the same green sandstone were prepared for testing, with a diameter of 2*R* = 100 mm, thickness of *B* = 30 mm, and crack length of *a* = 25 mm (i.e., *a*/*R* = 0.50). The tests were conducted with a ratio of half span to radius of 0.80 (i.e., *S*/*R* = 0.80), resulting in an approximate value of *Y* equal to 6.645. Six repeated fracture experiments were conducted using these SCB specimens, and a typical load–displacement curve is shown in Figure 15. The average peak load measured during these tests was approximately 1568 N. Consequently, the mode I fracture toughness *K*_IC_ of this sandstone, determined from Equation (3), was estimated to be around 0.97 MPa m^0.5^, which was significantly smaller than the true mode II fracture toughness *K*_IIC_.

The ratio of *K*_IIC_/*K*_IC_ of this sandstone ranges from 2.21 to 3.49 in the aforementioned four cases. If ignoring the influences of crack length and loading angle, the estimated average value of *K*_IIC_ is approximately 2.79 MPa m^0.5^. Consequently, the mean value of *K*_IIC_/*K*_IC_ of this sandstone is about 2.88. Yan et al. [4] reported an approximate ratio of *K*_IIC_/*K*_IC_ for a sandstone as 2.59. Additionally, Rao et al. [29] found that the range of *K*_IIC_/*K*_IC_ ratios varied from 2.40 to 3.20 for different rock types using the SBT method. It was reported by Yin et al. [71] that the *K*_IIC_/*K*_IC_ ratios for Fujian granite at room temperature varied from 2.74 to 3.24, as determined from the PTS specimen. Furthermore, the experimental findings of Bahrami et al. [39] indicated that the ratio of *K*_IIC_/*K*_IC_ for Bedretto granite ranged from 2.90 to 3.35 based on DCBD samples. These testing results obtained from DCFBD samples demonstrate strong consistency with previous studies.

## 5. Conclusions

The present study involved conducting extensive finite element analyses on DCFBD samples with varying crack lengths *α*, crack inclination angles *θ,* and load distribution angles *γ* to determine the SIFs *Y*_I_, *Y*_II_, and T-stress T*. The influences of *γ*, *α*, and *θ* on the *Y*_I_, *Y*_II_, and T* of DCFBD samples were systematically discussed. Moreover, a series of experiments were conducted on DCFBD samples to validate their shear (true mode II) fracture capability. The main conclusions are summarized as follows:

1. A significant number of numerical values for SIFs and T-stress were computed using FEM for DCFBD samples with *α* = 0.40~0.90, *θ* = 10°~45°, and *γ* = 5°~20°. These values play a crucial role in providing accurate descriptions of the compression-shear fracture tests conducted on this sample.
2. The effect of *γ* on *Y*_I_, *Y*_II_, and T* is remarkable for DCFBD samples with a smaller α. Nevertheless, it has been observed that for *α* ≥ 0.60 and *γ* ≤ 20°, the impact of *γ* on *Y*_I_ and *Y*_II_ can be disregarded; however, its effect on T* needs to be considered for DCFBD samples with a larger *θ*.
3. As *α* increases for *α* ≥ 0.60, the absolute value of *Y*_II_ increases while it decreases with increasing *θ*. Conversely, there is an opposite trend observed for *Y*_I_. Moreover, as *α* increases for *α* ≥ 0.60, the dimensionless T-stress T* tends to decrease while it increases with an increasing *θ*.
4. The experimental results show that the DCFBD samples with *θ* = *γ* = 10° and *θ* = *γ* = 15° can effectively achieve true mode II fracture for the two cases of relative crack lengths of 0.70 and 0.80. Furthermore, the average true mode II fracture toughness *K*_IIC_ of this sandstone is approximately 2.79 MPa m^0.5^, which significantly exceeds the mode I fracture toughness *K*_IC_ of 0.97 MPa m^0.5^, by a ratio of approximately 2.88.

## Figures and Tables

**Table 1:** The geometric dimensions of the DCFBD sample in finite element models.

Parameters	Values
Diameter	*D* = 100 mm
Dimensionless crack length	*α* = 0.40~0.90, with a increment of 0.05
Load distribution angle	*γ* = 5°, 10°, 15°, 20°
Crack inclination angle	*θ* = 10°~45°, with a increment of 5°

**Table 2:** Comparison of dimensionless SIFs and T-stress for the DCFBD samples.

Crack Inclination Angle and Load Distribution Angle	Dimensionless SIFs and T-Stress	Dimensionless Crack Length		
		*α =* 0.85	*α =* 0.75	*α =* 0.65
*θ = γ =* 10° [4]	*Y*_I_	−0.637	−0.740	−0.965
	*Y*_II_	−2.835	−2.468	−2.405
	T*	−6.025	−6.133	−6.215
*θ = γ =* 15° [4]	*Y*_I_	−0.874	−0.932	−1.126
	*Y*_II_	−2.761	−2.373	−2.255
	T*	−5.499	−5.450	−5.395
*θ = γ =* 10°	*Y*_I_	−0.632	−0.736	−0.959
	*Y*_II_	−2.850	−2.476	−2.406
	T*	−5.973	−6.049	−6.148
*θ = γ =* 15°	*Y*_I_	−0.870	−0.927	−1.118
	*Y*_II_	−2.772	−2.375	−2.258
	T*	−5.408	−5.374	−5.294

**Table 3:** Testing results obtained from DCFBD samples.

Relative Crack Length *α = a*/*R*	Loading Angle *θ = γ*/°	Dimensionless SIF *Y*_II_	Peak Load *P*/kN	Average Peak Load *P*_0_kN	True II Fracture Toughness *K*_IIC_/MPa m^0.5^
0.80	10	2.605	10.477	10.924	2.141
			11.572		
			10.022		
			9.987		
			12.564		
	15	2.519	14.899	13.799	2.614
			12.259		
			13.503		
			14.320		
			14.014		
0.70	10	2.418	18.774	17.768	3.023
			16.182		
			17.099		
			17.781		
			19.007		
	15	2.296	21.335	20.991	3.391
			23.134		
			22.715		
			19.183		
			18.592		

## Data Availability

The original contributions presented in this study are included in the article. Further inquiries can be directed to the corresponding author.

## Abbreviations

The following abbreviations are used in this manuscript:

DCBD	double-edge cracked Brazilian disc
DCFBD	double-edge cracked flattened Brazilian disc
DST	direct shear test
FEM	finite element method
PTS	punch-through shear
SBT	shear-box test
SCB	semi-circular bending
SIFs	stress intensity factors
ISRM	International Society for Rock Mechanics
